# Maximal Efficiency of PSII as a Marker of Sorghum Development Fertilized With Waste From a Biomass Biodigestion to Methane

**DOI:** 10.3389/fpls.2018.01920

**Published:** 2019-01-08

**Authors:** Zdzisława Romanowska-Duda, Mieczysław Grzesik, Regina Janas

**Affiliations:** ^1^Laboratory of Plant Ecophysiology, University of Łódź, Łódź, Poland; ^2^Department of Nursery and Seed Research, Research Institute of Horticulture, Skierniewice, Poland

**Keywords:** maximal efficiency of PSII, biomass biodigestion to methane, waste, fertilization, sorghum growth, physiological activity

## Abstract

The aim of experiments was to investigate a maximal efficiency of PSII, as a marker indicating growth, vigor, energetic value and physiological activity of sorghum fertilized with wastes from a biomass biodigestion to methane in a distillery integrated with a biogas plant using corn grains as substrate. The sorghum plants grown outdoor in different climate and in pots and in field were fertilized with different doses of the waste or Apol-humus – a soil improver and Stymjod – a nano-organic-mineral fertilizer. The maximal efficiency of PSII, in comparison with plant growth and health, chlorophyll content, gas exchange, activity of selected enzymes, element content in leaves and energetic value were studied. The wastes applied to soil resulted in increased maximal efficiency of PSII and the doses of 30 m^3^ ha^-1^ and 40–50 m^3^ ha^-1^ of the non-centrifuged and centrifuged ones, respectively, were most efficient. This enhancement was associated with the increased kinetics of plant growth, their health, fresh and dry biomass and physiological activity of plants as evidenced by activity of acid and alkaline phosphatase, RNase and dehydrogenase, as well as by gas exchange: net photosynthesis, transpiration, stomatal conductance, intercellular CO_2_ concentration and index of chlorophyll content in leaves. The fertilization with Apol-humus and Stymjod additionally increased maximal photochemical efficiency of PSII and plant development, biomass yield and physiological activity. The results indicate that waste from a biomass biodigestion to methane can be used as a natural fertilizer in sorghum crops and this ensures their recycling and environmental protection. The measurement values of maximal efficiency of PSII were proportionally to the vigor, growth and physiological activity of the plants. The obtained results indicate that the maximal efficiency of PSII in sorghum plants is a non-destructive method for defining the degree of growth and may be used as a marker of plant vigor and health, development and physiological activity expressed by gas exchange and activity of selected enzymes.

## Introduction

Management of waste from a biomass biodigestion to methane and its ecological use in plant production as fertilizers are among important issues in the world agriculture. This problem has become serious because the biogas plants are a prospective branch of energy production, as e.g., increase in electricity production from biogas in 2020 will amount to 125% as compared to 2010. Thus, the production of biogas is increasing and consequently the problem of economic management of the waste from a biomass biodigestion to methane has become very important from economic and environmental point of view. Given the environmental risks and benefits, it is most rational to use these wastes in agriculture (Alburquerque et al., 2012; Jasiulewicz and Janiszewska, 2013; Łagocka et al., 2016), probably to a greater extent than sewage sludge, which may contain toxic substances and should be refined before use in plant crops (Fang et al., 2017; Kołecka et al., 2017). World literature available on these issues, especially concerning agricultural management of waste from corn grains biodigestion to methane, is hard to find and in the majority of cases it refers to the waste produced by specific biogas plants and the raw materials used there (Lukehurst et al., 2010; Nakamura et al., 2014; Lalak et al., 2015).

The problem in the waste application in agriculture is a quick and reliable assessment of the effects of plant fertilization that would enable rational management of this treatment. For this reason, technologists are looking for physiological methods that immediately show the reaction of the plants to different treatments, which enables quick cultivation decisions to be made in the growing season without waiting for the completing plant growth in autumn. One of such checked tests is the evaluation of key for growth enzyme activity (acid and alkaline phosphatase, RNase, nitrate reductase, dehydrogenase), index of chlorophyll content and gas exchange in leaves (net photosynthesis, transpiration, stomatal conductance, intercellular CO_2_ concentration (Górnik et al., 2008; Grzesik and Romanowska-Duda, 2014, 2015; Piotrowski et al., 2016; Grzesik et al., 2017a,b). Kalaji M.H. et al. (2014), Kalaji et al. (2018) indicated that chlorophyll fluorescence parameters may be taken into account as an interesting new indicator which can measure most types of plant stress and perhaps their vigor and thus they published a practical information on the hardware, methodology and the hands on application of this technology. Chlorophyll fluorescence measurements allow the recognition of changes in the general bioenergetic state of the photosynthetic apparatus. Moreover, such measurements relate, directly or indirectly, to all stages of light-dependent photosynthetic reactions, including water splitting, electron transport, and pH gradient formation across the thylakoid membrane and ATP synthesis (Kalaji et al., 2012). The chosen fluorescence parameters can give insights into the ability of plants to tolerate environmental stresses and into the extent to which those stresses have damaged the photosynthetic apparatus. By measuring the intensity and nature of this fluorescence, plant ecophysiology can be investigated (Lichtenthaler et al., 1986; Żebrowska and Michałek, 2014). Strasser et al. (2000) suggested six parameters which can be used in screening tests: F_0_/F_m_, (dV/dt)_0_, VJ, V_I_, t_Fmax_ and S_m_. They describe that F_0_/F_m_, (dV/dt)_0_ and VJ refer to the structure and function of PSII while V_I_, t_Fmax_ and S_m_ concern the activity of the electron transport chain (ETC) beyond Q_A_. Among parameters of chlorophyll fluorescence, the maximum PSII efficiency, as given by F_v_/F_m_ in dark-adapted leaves is more often used in research (Su et al., 2015) probably because it is more frequently used and commonly available from early pulse amplitude modulated (PAM) fluorimeters (Osinga et al., 2012). The ratio-metric normalization of F_v_/F_m_ also facilitates ease of results interpretation. The F_v_/F_m_ is calculated as (F_m_ - F_o_)/F_m_ when F_o_ means a (minimal) fluorescence level of dark-adapted sample when all reaction centers of the photosystem II are open and F_m_ is a (maximal) fluorescence level of dark-adapted sample when a high intensity pulse has been applied and the all reaction centers of the photosystem II are closed (Kitajima and Butler, 1975).

Chlorophyll fluorescence has been found as the genetic marker of barley productivity (Planchon et al., 1989). Jalink et al. (1998) and Górnik et al. (2013) indicated that it can be used for assessment of maturation advancement of cabbage and coriander seeds, taking into consideration that their degreening process is directly related with the amount of chlorophyll in seed coats, which was monitored by measuring of maximal efficiency of PSII. Górnik and Lachuta (2017) demonstrated that the changes in maximal photochemical efficiency of PSII (F_v_/F_m_) in sunflower seedlings was related to their growth, which was reduced by previous chilling and increased by subsequent priming of seeds, sown then after these treatments to obtain seedlings subjected to assessments of physiological activity. Chlorophyll fluorescence was also proposed as a marker of the plant reactions to herbicides mechanism of action (Dayan et al., 2012), cellular damage by glyphosate herbicide in *Raphanus sativus* L. plants (Silva et al., 2014) and it was used in physiological evaluation of strawberry varieties (Żebrowska and Michałek, 2014). Available literature indicates fragmentarily on changes in maximal efficiency of PSII under the influence of selected plant treatments. Checking the applicability of maximal efficiency of PSII measurements as a marker of plant responses to different fertilization methods requires further extensive research using different cultivation conditions and fertilization methods, as well as comparison to the tests of vigor and plant reaction recognized in literature so far. To the best our knowledge, the use of maximal efficiency of PSII as the marker of energy plant vigor, including sorghum, fertilized with waste from a biomass biodigestion to methane and selected biostimulators, as well as its comparison to a wide range of previously used plant vigor evaluation tests, was not studied yet, despite the urgent need to develop a quick and non-invasive methods for assessing plant response to fertilization and making rapid decisions concerning their cultivation before completing growth.

The purpose of this work was to investigate the suitability of maximal efficiency of PSII, as the marker indicating growth, vigor, energetic value and physiological activity of sorghum fertilized with wastes from a corn grain biodigestion to methane, supplemented with new generation biostimulators, Apol-humus and Stymjod, and its usefulness in monitoring of waste applicability in sorghum crops.

## Materials and Methods

### Plants, Waste From a Biomass Biodigestion to Methane, Biostimulators and Soil

The commercial seeds of sorghum (*Sorghum bicolor* L.) ‘Rona 1’ obtained from the breeding company “Kutnowska Hodowla Buraka Cukrowego” in Poland, were used in experiments. The chosen variety is cultivated for silage with a lower content of non-digestible fiber and as an energy plant. It gives high yields on weak soils of IV and V bonitation class and in conditions of shortage precipitation. Thus, it is an alternative to maize that requires more fertile and moistening soils, which assurance is difficult in a changing climate.

The liquid, non-centrifuged and centrifuged wastes from a corn grain biodigestion to methane, were supplied by the distillery integrated with the biogas plant in Piaszczyna (Gamawind Sp. z o.o., Poland), which produce alcohol and biogas. The tested waste were rich in most of macro- and microelements necessary for plant growth. The non-centrifuged waste contained more nutrients than the centrifuged one (Table 1).

**Table 1:** Content of macro- and micronutrients and physical properties of liquid non-centrifuged and centrifuged wastes from a biomass biodigestion to methane, Stymjod, Apol-humus, and peat substrate in 5-l pots and in podzolic soil in field.

	Waste not centrifuged	Waste centrifuged	Stymjod^1^	Apol -humus	Substrate in pots	Soil in field
Elements	[mg L^-1^]	[mg L^-1^]	[mg L^-1^]	[mg L^-1^]	[mg L^-1^ d.w.]	[mg L^-1^ soil]
**Basic analyze**						
N-NO_3_^-^	<1.0	<1.0	1231	4.30	10.8	44.0
N-NH_4_^+^	2460	670	–	10.9	118.0	126.0
P	276	184	6652	15.7	71.0	87.0
K^+^	977	855	62720	20.0	60.3	67.0
Ca^+2^	290	174	943	466	1030.0	4840.0
Mg^+2^	113	61.3	11570	71	51.0	53.0
Na^+^	509	514	–	–	–	–
Cl^-^	643	662	–	–	–	–
SO_4_^-2^	140	107	–	–	–	–
**Microelements**						
Fe	9.0 54	6.768	18.9	142	–	–
Mn	0.338	0.270	886	5.98	–	–
Cu	0.189	0.096	682	0.89	–	–
Zn	0.968	0.637	1476	2.42	–	–
B	3.357	3.116	576	0.94	–	–
% d.w. eight	1.5	0.5	–	–	–	–
**Physical properties**						
pH	7.6	7.2	–	12.0	5.0	7.2


*^*1*^Stymjod contained also: humic acids (3.3% C kg^-*1*^), organic substances including amino acids (56.8%), aqueous iodine concentrate I_*n*_^+^ 0.0025%.*

Apol-humus, the ecological soil improver was supplied by the producer, Poli-Farm Sp. z o.o., Poland, while Stymjod, the nano-organic-mineral fertilizer was obtained from manufacturer PHU Jeznach Sp. J., Poland (Jeznach, 2015) (Table 1).

The sphagnum peat substrate, used for filling of 5-l pots and containing nutrients listed in Table 1, were supplied by Alonet Substrate KS (Latvia).

The soil on the field plots (3 × 3 m each) has been classified as podzolic and contained nutrients listed in Table 1.

### Treatments and Experimental Design

In order to check the usefulness of the maximum PSII efficiency, compared to other tests used so far, in various cultivation conditions, research was performed and plants were cultured outdoor in two places 400 km apart, slightly differing in climate, one in central Poland where pots (filled with peat substrate) were used and the other in northern Poland in the fields, in podzolic soil. In central Poland, temperature in July fluctuates from 8 to 32°C, annual average precipitation is 528.3 mm and dry air, while in the north these parameters are respectively from 11 to 21°C, 655 mm and humid air from the nearby Baltic Sea. In July, when physiological properties of plants were assessed, the temperature in central Poland was much over 30°C (nearby Baltic Sea less of 1–3°C) in sun and through the all month a more sunny and less windy days were noted than in the north. All cultivation treatments and plant measurements were carried out at similar times in experiments performed in pots and in the field.

In the middle of April, the peat substrate in each 10 pots forming a plot and the podzolic soil in field plots were fertilized with waste dosages and biostimulants, as follows:

(I) non-centrifuged liquid waste from a biomass biodigestion to methane (E) at dosages of 10, 20, 30, and 40 m^3^ ha^-1^,

(II) centrifuged liquid waste (C) at dosages of 20, 30, 40, 50, and 60 m^3^ ha^-1^,

(III) pot and field plots fertilized with the most favorable doses of waste (E 30 m^3^ ha^-1^ and C 40 m^3^ ha^-1^) were additionally fertilized with Apol-humus (AH, 10 L ha^-1^), and also with applied together Apol-humus (10 L ha^-1^) to soil and Stymjod 1.5% (5 L ha^-1^) used as twice leaf sprayings.

The applied waste and Apol-humus were mixed with soil directly after fertilization.

The fertilized differently pot plots and field plots were situated randomly and in three repetitions per treatment.

In the middle of May, seeds of sorghum were sown to (fertilized previously with waste and Apol-humus) peat substrate, one per pot, and to soil in field, 175 ones on plot, in rows 35 cm apart, and the distance between seeds in row 12 cm, according to the common practice.

Not fertilized plants and watered or sprayed with water at the same doses as waste or biostimulants, were served as control. Additional controls were the treatments of soil with Apol-humus and plants with Stymjod, which were used also to check whether they increase the plant’s response to used fertilization, as suggested by their producers. The applied dose range (allowing their optimization) was selected on the previous laboratory research in modernized Phytotoxkit plates, which showed that centrifuged waste should be used in a larger quantity than not subjected to centrifugation.

### Assessments of Plant Development, Physiological Activity and Chemical Properties

#### Non-destructive Measurements

Non-destructive measurements of physiological activity (maximal efficiency of PSII, gas exchange, index of chlorophyll content, mycological analyzes) and destructive ones (enzymatic activity, nutrient content in leaves) were carried out on fully developed leaves located at the highest level on the plants cultivated in pots and field. In each treatment, one leaf from each of the five plants or their 100 g for nutrient content evaluation, was used for assessment at the end of July at temperature over 30°C in sun and air humidity of about 50%.

Chlorophyll fluorescence were assessed by the measurements of the maximum efficiency of photosystem II (PSII), as given by F_v_/F_m_ in dark-adapted leaves, with a pulse modulated Fluorescence Monitoring System (FMS-1; Hansatech Instruments Ltd., Norfolk, United Kingdom) operated in the F_v_/F_m_ mode, according to the instruction of producer. For measurement the fully developed leaves and placed in the highest part of plant were taken. Before assessment, the leaves were kept in dark for 20 min in order to initiate all reaction centers to open and minimize physiological processes associated with the energization of the thylakoid membrane. According to apparatus manufacturer, the fiber optic of the FMS-1 was situated by using the PPF/temperature leaf clip at a 60° angle from the leaf upper surface. This did not significantly interfere with PPF distribution at the leaf surface, yet it allowed delivery of a saturation pulse of actinic light and detection of fluorescence signals (Cheng et al., 2000). The space between the surface of leaves and fiber optic was constant for all evaluations. The maximum fluorescence (F_m_), and variable fluorescence F_v_, given as F_m_ – F_o_, were assessed in the leaves adapted to darkness using leaf clips. The initial fluorescence (F_o_) was evaluated at PPFD < 0.05 μmol m^-2^ s^-1^, following by a saturating pulse to determine the maximum fluorescence emission in the absence (F_m_) of quenching was 3000 μmol m^-2^ s^-1^. The intensity of saturation pulses to evaluate a maximum fluorescence emission in the absence (F_m_) of quenching was 1800 μmol m^-2^ s^-1^. Maximum photochemical efficiency of PSII was estimated by the calculation: F_v_/F_m_ = (F_m_ - F_o_)/F_m_ (Kalaji M.H. et al., 2014; Górnik and Lachuta, 2017).

Height of shoots was measured every month during vegetative season, from the soil up to the plant top (Grzesik et al., 2017b).

Activity of gas exchange, reflected by net photosynthesis rate (Pn; μm CO_2_ × m^-2^ × s^-1^), transpiration (E; mmol H_2_O × m^-2^ × s^-1^), stomatal conductance (Gs; mmol H_2_O^-1^ × m^-2^ × s^-1^), intercellular CO_2_ concentration (Ci; μmol CO_2_ air × mol^-1^), was measured using the portable photosynthesis measurement system TPS-2 (PP Systems, United States) (Grzesik et al., 2017a,b).

Index of chlorophyll content (in SPAD units) in leaves was evaluated using Minolta SPAD-502 chlorophyll meter (Konica Minolta, Japan) (Pacewicz and Gregorczyk, 2009; Grzesik and Romanowska-Duda, 2014; Grzesik et al., 2017a,b).

The infestation of plants by pathogenic fungi and their quality and quantity were evaluated after harvest of infected leaves (one from each of 10 plants) and subsequent their incubation for 7-day in humid cameras. The fungi were then transplanted from leaves into selective media, on which their species composition and quantity was identified using light microscopy (Leica) and available identification keys (Mehta et al., 2014). The percentage of plants infested with pathogenic fungi was assessed, taking into account 10 plants in pot experiment and 175 ones in the field.

#### Enzyme Activity, Nutrient Content and Biomass Yield

Activities of alkaline (pH 7.5) (EC 3.1.3.1) and acid (pH 6) (EC 3.1.3.2) phosphatase [U g^-1^ (FM) min^-1^] and RNase (EC 3.1.27.5) [U g^-1^(FM) min^-1^] in leaves were examined using the methods elaborated by Knypl and Kabzińska (1977).

Total dehydrogenase activity (EC 1.1.1.-) [mg (formazan) g^-1^ (leaf fm] was evaluated, by the procedure described by Górnik and Grzesik (2002) and Grzesik et al. (2017a), using spectrophotometer UVmini-1240 (Shimadzu, Japan) for formazan determination at a wavelength of 480 nm. Enzyme activities were calculated on the basis of fresh mas (fm).

The weight of fresh (directly after harvest) and dry (after drying for 3 days at 130°C) biomass, of 5 plants per repetition and then calculated for 1 plant presented as average for treatment, was evaluated at the end of November (Grzesik et al., 2017b).

The content of elements in leaves, Stymjod, Apol-humus, peat substrate in pots and podzolic soil in field was evaluated in a certified laboratory at Research Institute of Horticulture in Skierniewice (Poland), using standard procedures. The material was concentrated by evaporation of excess water and then mineralized in a microwave oven “Etho-1” from Milestone, Italy. In the solution after mineralization, quantity and composition of elements were determined using a plasma spectrometer (ICP) model DV2000 from Perkin-Elmer, United States. Different wavelengths characteristic of a given element were used for the determination of elements. The total nitrogen content was determined by the Kjeldahl method after mineralization in concentrated sulfuric acid with the addition of a catalyst. The determination of the nitrogen content was carried out using a titration (Antweiler et al., 1996). Energetic value of plants was estimated by Carbochem, a certified laboratory (Poland) using Polish assessment standards listed in Table 8 (Grzesik et al., 2017b).

### Statistical Analysis

All experiments were performed in two series in central and northern Poland, in three replicates for each treatment. Within each series, each repetition was set up in a completely randomized block design. The obtained data, given as means from replicates, were processed applying analysis of variance (ANOVA I), by Statistica 12. The means of chosen parameters were grouped employing the Dunett’s test and the contrast between control sample and the remaining samples was used at α = 0.05 significance level. The confidence limit at 0.95 was shown in all drawings. The data in tables marked with the same letters within a column are not significantly different, according to Newman – Keuls multiple range test at an alpha level of 0.05. To predict sorghum yields based on chlorophyll fluorescence, linear regression was applied.

## Results

### Non-destructive Measurements in Pot and Field Experiments

**FIGURE 1:**
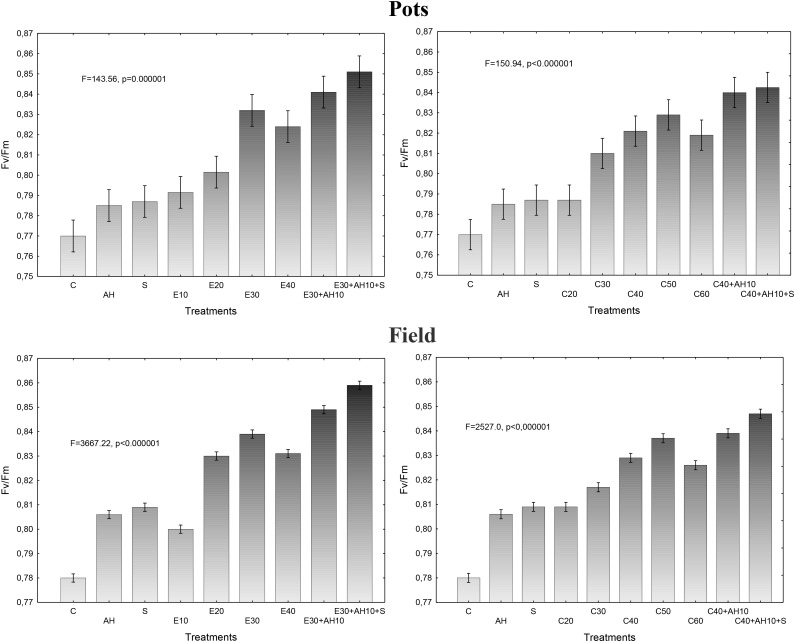
Maximum PSII efficiency in sorghum leaves cultivated in pots and field and fertilized with liquid waste from a biomass biodigestion to methane, non-centrifuged (E 10–40 m^3^ ha^-1^) and centrifuged (C 20–60 m^3^ ha^-1^), Apol-humus (AH; 10 L ha^-1^) and Stymjod (S; 5 L ha^-1^).

Maximum efficiency of photosystem II (PSII) measurements, as given by F_v_/F_m_ in dark-adapted sorghum leaves in pot experiments, were closely related to the plant fertilization methods and showed that all used treatments with both, waste and biostimulants increased this parameter. However, the increase in the maximal efficiency of PSII depended on the fertilizers, their doses and method of application. The non-centrifuged waste applied to soil at the dose of 10–40 m^3^ ha^-1^ and the centrifuged one at 20–60 m^3^ ha^-1^ significantly increased the maximal efficiency of PSII, which showed also that the most profitable were their doses of 40 m^3^ and 40–50 m^3^, respectively. The positive impact of both forms of waste, shown by this parameter, was enhanced by the previous application of the soil improver Apol-humus at the dose of 10 L ha^-1^ and moreover, to higher degree, by additional twofold plant spraying with nano-organic-mineral fertilizer Stymjod, at 2-week interval, at the concentration of 1.5% (5 L ha^-1^) (Figure 1).

Established dependencies between the doses of non-centrifuged and centrifuged waste, supplemented with biostimulants and the maximum efficiency of PSII in the pot investigations were confirmed in field experiments conducted in more fertile podzolic soil and under slightly other climatic conditions of northern Poland nearby Baltic Sea than in center of the country. Due to more favorable soil conditions in field for plant development, the maximal efficiency of PSII was proportionally higher in plants cultivated in field, than in 5-l pots, as it was shown in all treatments (Figure 1).

**FIGURE 2:**
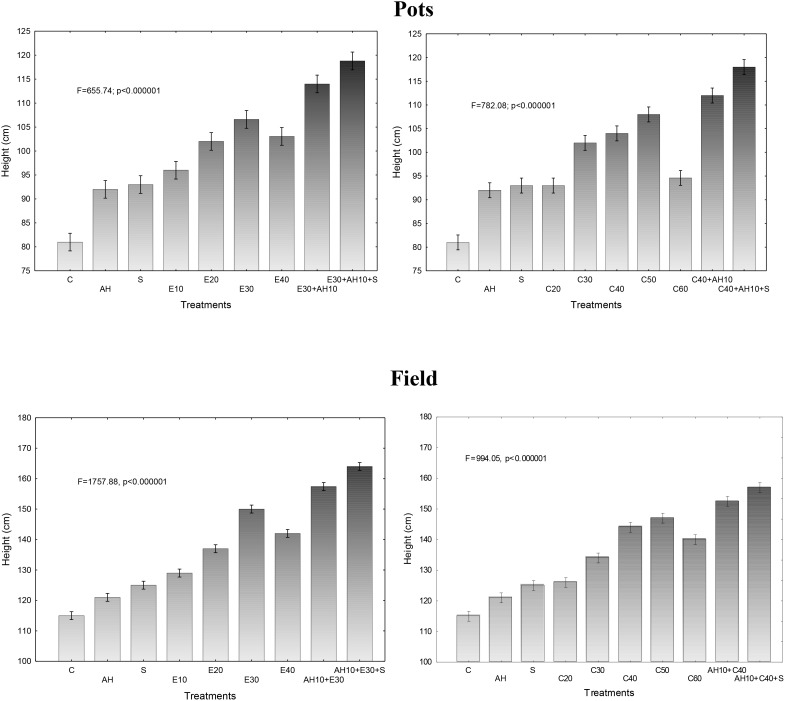
Final height of sorghum plants cultivated in pots and field and fertilized with liquid waste from a biomass biodigestion to methane, non-centrifuged (E 10–40 m^3^ ha^-1^) and centrifuged (C 20–60 m^3^ ha^-1^), Apol-humus (AH; 10 L ha^-1^) and Stymjod (S; 5 L ha^-1^).

Plant growth kinetics in 5-l pots, exhibited by results of the shoot height measurements every month during all vegetative season, were dependent on the waste dose and whether they were used alone or together with biostimulators, similarly as it was found in the study of maximal efficiency of PSII. Since the dependencies between effects of treatments were similar throughout the growing season, the presented results in Figure 2 show the results of final height measurement in November, as average of the measured plants in each repetition. Correspondingly, as the results of test described above showed, a non-centrifuged waste applied at the dose of 10–40 m^3^ ha^-1^ and the centrifuged one of 20–60 m^3^ ha^-1^ accelerated growth of plants. Similarly, the higher growth kinetics were observed when the doses were 30 m^3^ ha^-1^ and 40–50 m^3^ ha^-1^ for non-centrifuged and centrifuged wastes, respectively. Correspondingly, as maximal efficiency of PSII analyze showed also, application of Apol-humus to soil and Stymjod to plants increased their growth, as compare to control if they were used alone, or in comparison to optimal dosages of waste if these biostimulants were applied together with them (Figure 2).

The found relations between the doses of waste and biostimulators and the growth of plants in pot studies were confirmed in field experiments. Plants cultivated in the field in all experimental variants were proportionally higher than those grown in 5-liter pots. Similarly, they maintained the same relationship between the methods of fertilization with waste and biostimulators and the growth of plants that were found in the tests discussed above (F_v_/F_m_) (Figure 2).

The observed favorable changes in maximal efficiency of PSII comparable to changes in plant growth were confirmed by the enhanced proportionally activity of gas exchange and increased index of chlorophyll content in leaves, which were the consequence of the application of waste and stimulants in 5-l pots. Similarly, as the results of presented above two tests showed, the used waste and biostimulants increased activity of gas exchange processes and index of chlorophyll content in leaves, measured at high temperature, over 30°C in sun and in low air humidity about 50%. As shown in Table 2, the wastes increased the index of chlorophyll content, net photosynthesis, transpiration, stomatal conductance and decreased intercellular CO_2_ concentration, inversely proportional to mentioned gas exchange parameters. The dependencies between the impacts of the used waste doses and gas exchange features or index of chlorophyll content were similar to these observed between waste doses, plant height and maximal PSII efficiency. As they show, the non-centrifuged waste increased gas exchange parameters more than the centrifuged one used in the same doses and their application in doses of 30 m^3^ ha^-1^ and 40–50 m^3^ ha^-1^ respectively, together with biostimulants were most efficient (Figures 1, 2 and Table 2).

**Table 2:** Parameters of gas exchange and index of chlorophyll content in sorghum leaves of plant cultivated in pots and fertilized with liquid, non-centrifuged and centrifuged waste from a biomass biodigestion to methane, Apol-humus and Stymjod.

	Net photosynthesis	Transpiration	Stomatal conductance	Concentration of intercellular	Index of chlorophyll
Applied waste	[*μ*m CO_2_ m^-2^s^-1^]	[mmol H_2_O m^-2^s^1^]	[mmol H_2_O^-1^ M^-2^ s^-1^]	CO_2_[*μ*mol CO_2_ air mol^-1^]	content SPAD
**Control and treatment with biostimulators**					
Control	3.6 a	0.83 a	218.0 a	380.0 h	20.4 a
Apol-humus	3.9 b	1.00 b	318.0 b	369.0 g	21.1 b
Stymjod	3.9 b	1.0 2 b	320.0 b	368.0 g	22.1 c
**Substrate fertilized with non-centrifuged waste (E) at doses of 10–40 m^3^ ha^-1^ and Apol-humus (AH; 10 L ha^-1^) and plant spraying with**					
**Stymjod (S; 5 L ha^-1^)**					
E10	5.8 g	1.08 b	340.0 cd	362.0 fg	24.0 e
E20	6.9 h	1.09 b	366.0 e	350.0 de	25.1 g
E30	7.3 ij	2.00 f	400.0 g	347.0 cd	26.1 ij
E40	7.2 i	1.50 de	359.0 e	355.0 ef	25.9 i
E30+AH10	7.5 jk	2.13 h	420.0 h	335.0 b	26.3 j
E30+AH10+S	7.7 k	2.30 i	448.0 i	326.0 a	27.0 k
**Substrate fertilized with centrifuged waste (C) in doses of 20–60 m^3^ ha^-1^ and Apol-humus (AH; 10 L ha^-1^) and plant spraying with**					
**Stymjod (S; 5 L ha^-1^)**					
C20	3.9 b	1.31 c	320.0 b	369.0 g	23.0 d
C30	4.0 bc	1.42 d	330.0 bc	352.0 de	25.3 gh
C40	4.5 ef	1.53 e	350.0 d	349.0 de	25.6 h
C50	4.3 de	1.30 c	325.0 b	350.0 de	25.5 h
C60	4.2 cd	1.03 b	320.0 b	367.0 g	24.5 f
AH10+C40	4.7 f	1.53 e	368.0 e	340.0 bc	25.9 i
AH10+C40+S	6.9 h	1.70 f	379.0 f	335.0 b	26.3 j


*The data marked with the same letters within a column are not significantly different, according to Newman–Keuls multiple range test at an alpha level of 0.05.*

The examined dependences, found between all fertilization variants and the presented measurements in field conditions, closely coincided with the relationships found in the pot experiment. Plants growing in field, in more favorable soil conditions and at slightly lower temperature than in central Poland, showed slightly higher value of net photosynthesis, transpiration and stomatal conductance than in pots, however, the relationship between all these mentioned parameters and applied waste doses were similar (Tables 2, 3).

**Table 3:** Parameters of gas exchange and index of chlorophyll content in sorghum leaves of the plants cultivated in field and fertilized with liquid, non-centrifuged and centrifuged waste from a biomass biodigestion to methane, Apol-humus, and Stymjod.

Applied waste	Net photosynthesis	Transpiration	Stomatal conductance	Intercellular concentration of	Index of chlorophyll
	[μm C02 m^-2^s^-1^]	[mmol H2O m^-2^s^-1^]	[mmol H_2_O^-1^ M^-2^s^-1^]	CO_2_[μmol CO_2_ air mol^-1^]	content SPAD
**Control and treatment with biostimulators**					
Control	4.4 a	0.91 a	229.0 a	375.0 g	20.8 a
Apol-humus	4.7 b	1.05 b	241 b	367 f	21.5 b
Stymjod	4.7 b	1.08 b	245 b	360 f	22.4 c
**Soil fertilized with non-centrifuged waste (E) at doses of 10–40 m^3^ ha^-1^ and Apol-humus (AH; 10 L ha^-1^) and plant spraying with**					
**Stymjod (S; 5 L ha^-1^)**					
E10	5.9 fg	1.09 b	352.0 d	360.0 f	24.3 e
E20	6.9 h	1.49 d	368.0 c	348.0 de	25.4 gh
E30	7.4 i	2.03 fg	408.0 gh	341.0 bcd	26.3 kl
E40	7.1 h	1.82 e	388.0 f	350.0 e	25.9 ij
E30+AH10 E10	7.7 j	2.16 h	428.0 i	334.0 b	26.61
E30+AH10+S	8.0	2.35 i	454.0 j	321.0 a	27.3 m
**Soil fertilized with centrifuged waste (C) in doses of 20–60 m^3^ ha^-1^ and Apol-humus (AH; 10 L ha^-1^) and plant spraying with**					
**Stymjod (S; 5 L ha^-1^)**					
C20	4.8 be	1.33 c	246.0 b	361.0 f	23.4 d
C30	5.2 de	1.81 e	375.0 e	350.0 e	25.6 hi
C40	5.4 e	1.94 f	389.0 f	342.0 cd	25.4 gh
C50	5.0 cd	1.52 d	349.0 d	344.0 de	25.1 fg
C60	4.7 b	1.33 c	331.0 c	361.0 f	24.8 f
AH10+C40	5.7 f	2.05 g	401.0 g	336.0 c	26.2 jk
AH10+C40+S	6.1 g	2.17 h	413.0 h	327.0 d	26.5 kl


*The data marked with the same letters within a column are not significantly different, according to Newman–Keuls multiple range test at an alpha level of 0.05.*

Plant infestation by pathogenic mycoflora were similar in pot tests and field conditions. The infestation of plants grown in pots and field by pathogenic mycoflora was reduced by applied waste and biostimulants. As shown in Tables 4, 5, eight species of pathogenic fungi were isolated from the infected leaves. Percentage of all isolated pathogenic fungi in relation to total isolates, and the percent of infected plants in comparison to control, decreased under influence of all fertilization methods. The highest decrease in fungi infestation was observed after the use of waste doses of 30 m^3^ ha^-1^ (non-centrifuged) and 40 m^3^ ha^-1^ (centrifuged) and biostimulators (Tables 4, 5). The reduction of plant infestation by pathogenic fungi was inversely proportional to the increase in F_v_/F_m_ (Figure 1 and Tables 4, 5).

**Table 4:** Species composition and percentage of pathogenic fungi on infested leaves in relation to total isolates and the percent of infected plants, as effected by sorghum plant fertilization with waste from a biomass biodigestion to methane (E), Apol-humus (AH) and Stymjod (S) in pots.

Doses of non-centrifuged waste (m^3^ ha^-1^)									

Pathogenic fungi	Control	AH	S	E10	E20	E30	E40	E30+ AH 10	E30+ AH 10 +S
**Percentage of pathogenic fungi on infested leaves in relation to total isolates**									
*Colletotrichum sublineolum*	8.9 c	8.1 c	6.2 b	8.1 c	6.2 b	5.7 ab	5.4 ab	5.1 a	5.0 a
*Setosphaeria turcica*	5.8 c	5.3 c	3.3 b	5.3 c	3.2 b	2.1 a	2.3 a	2.0 a	1.9 a
*Fusarium* ssp.	3.5 d	3.1 cd	2.7 c	3.1 cd	2.7 c	0.0 a	1.3 b	0.0 a	0.0 a
*Puccinia sorghi*	2.4 d	2.1 c	2.1 c	2.1 c	0.5 b	0.0 a	0.0 a	0.0 a	0.0 a
*Phoma* sp.	1.5 d	1.0 c	1.0 c	1.0 c	0.5 b	0.0 a	0.0 a	0.0 a	0.0 a
*Alternaria* sp.	7.0 c	5.0 b	5.0 b	6.3 c	5.0 b	3.4 a	3.2 a	3.1 a	2.8 a
*Verticillium* sp.	1.1 d	0.6 c	0.6 c	0.6 c	0.2 b	0.0 a	0.2 a	0.0 a	0.0 a
*Pythium* sp.	1.0 c	0.7 b	0.7 b	0.7 b	0 a	0.0 a	0.0 a	0.0 a	0.0 a
**Percent of infected plants**									
Infested plants (%)	19.2 f	15.0 e	15.0 e	15.2 e	10.3 cd	9.3 bc	11.8 d	8.3 ab	6.3 a


*The data marked with the same letters within a lines are not significantly different, according to Newman–Keuls multiple range test at an alpha level of 0.05.*

**Table 5:** Species composition and percentage of pathogenic fungi on infested leaves in relation to total isolates and the percent of infected plants, as effected by sorghum plant fertilization with waste from a biomass biodigestion to methane (E), Apol-humus (AH) and Stymjod (S) in field.

Doses of non-centrifuged waste (m^3^ ha^-1^)									

Pathogenic fungi	Control	AH	S	E10	E20	E30	E40	E30 + AH 10	E30 + AH 10 + S
**Percentage of pathogenic fungi on infested leaves in relation to total isolates**								
*Colletotrichum sublineolum*	9.1 c	6.5 b	6.3 b	8.5 c	6.4 b	5.9 ab	5.6 ab	5.0 a	5.0 a
*Setosphaeria turcica*	5.9 c	3.5 b	3.3 b	5.5 c	3.5 b	2.1 a	2.5 a	2.1 a	2.0 a
*Fusarium* ssp.	3.7 d	2.9 c	2.8 c	3.2 cd	2.9 c	0.9 a	1.4 b	0.0 a	0.0 a
*Pucinia sorghi*	2.7 c	0.7 b	0.6 b	2.5 c	0.5 b	0.0 a	0.0 a	0.0 a	0.0 a
*Phoma* sp.	1.7 d	1.5 c	0.8 b	1.5 c	0.7 b	0.1 a	0.2 a	0.0 a	0.0 a
*Alternaria* sp.	7.4 c	5.9 b	5.4 b	6.6 c	5.4 b	3.5 a	3.3 a	3.3 a	2.9 a
*Verticillium* sp.	1.5 d	0.5 b	0.4 b	0.8 c	0.4 b	0.1 a	0.2 a	0.0 a	0.0 a
*Pythium* sp.	1.0 c	0.8 b	0.7 b	0.8 b	0.2 a	0.0 a	0.0 a	0.0 a	0.0 a
**Percent of infected plants**								
Infested plants (%)	21.1 e	16.6 d	15.9 d	15.6 d	10.8 b	9.4 b	12.3 c	8.7 ab	6.5 a


*The data marked with the same letters within a lines are not significantly different, according to Newman–Keuls multiple range test at an alpha level of 0.05.*

### Enzyme Activity and Physical Properties and Yield of Biomass in Pot and Field Experiments

Applied doses of waste used separately or together with biostimulators caused a positive impact on the activity of selected enzymes (acid and alkaline phosphatase, RNase, dehydrogenases) that have a key impact on plant development. The changes in the activity of acid and alkaline phosphatase, RNase and dehydrogenase were closely related to the positive effect of fertilization with various doses of waste and biostimulants, and were proportional to the maximal efficiency of PSII and measurements of other studies. Here also, the more increased enzyme activity was affected by non-centrifuged and centrifuged waste application to the soil at a dose of 30–40 m^3^ ha^-1^ and 40–50 m^3^ ha^-1^, respectively. Their supplementation with Apol-humus Stymjod additionally improved this beneficial effect, similarly as it was found during maximal PSII efficiency measurements (Tables 6, 7).

**Table 6:** Activity of selected enzymes in leaves of sorghum plants cultivated in pots and fertilized with liquid, non-centrifuged and centrifuged waste from a biomass biodigestion to methane, Apol-humus and Stymjod.

	Phosphatase (pH = 6.0)	Phosphatase (pH = 7.5)	RNase	Total dehydrogenases
Treatment	[U g^-1^ f.w.]	[U g^-1^ f.w.]	[U g^-1^ f.w.]	[mg formazan × g leaf^-1^]
**Control and treatment with biostimulators**				
Control	0.57 a	0.19 a	2.60 a	0.50 a
Apol-humus	0.632 ab	0.23 b	2.9 b	0.62 b
Stymjod	0.62 ab	0.23 b	3.0 b	0.61 b
**Substrate fertilized with non-centrifuged waste (E) at doses of 10–40 m^3^ ha^-1^ and Apol-humus (AH; 10 L ha^-1^) and plant spraying with**				
**Stymjod (S; 5 L ha^-1^)**				
E10	0.64 bc	0.23 b	3.10 b	0.63 bc
E20	0.69 cde	0.27 cd	3.32 cd	0.68 cde
E30	0.75 fg	0.31 e	3.50 d	0.75 fg
E40	0.73 def	0.28 de	3.42 d	0.70 def
E30+AH10	0.82 hi	0.35 f	3.89 e	0.82 h
E30+AH10+S	0.89 j	0.39 gh	4.29 f	0.89 i
**Substrate fertilized with centrifuged waste (C) in doses of 20–60 m^3^ ha^-1^ and Apol-humus (AH; 10 L ha^-1^) and plant spraying with**				
**Stymjod (S; 5 L ha^-1^)**				
C20	0.62 ab	0.23 b	2.96 b	0.62 b
C30	0.68 cd	0.27 cd	3.14 bc	0.69 de
C40	0.74 ef	0.31 e	3.50 d	0.76 g
C50	0.69 cde	0.28 de	3.39 d	0.71 efg
C60	0.61 ab	0.24 bc	3.03 b	0.65 bcd
AH10+C40	0.80 gh	0.36 fg	3.88 e	0.82 h
AH10+C40+S	0.86 ij	0.40 h	4.28 f	0.88 i


*The data marked with the same letters within a column are not significantly different, according to Newman–Keuls multiple range test at an alpha level of 0.05.*

**Table 7:** Activity of selected enzymes in leaves of sorghum the plants cultivated in field and fertilized with liquid, non-centrifuged and centrifuged waste from a biomass biodigestion to methane, Apol-humus and Stymjod.

	Phosphatase	Phosphatase	RNase	Total dehydrogenases
Treatment	(pH = 6.0) [U g^-1^ f.w.]	(pH = 7.5) [U g^-1^ f.w.]	[U g^-1^ f.w.]	[mg formazan × g leaf^-1^]
**Control and treatment with biostimulators**				
Control	0.60 a	0.22 a	2.70 a	0.59 a
Apol-humus	0.65 bc	0.25 b	2.94 b	0.64 b
Stymjod	0.65 bc	0.25 b	2.97 b	0.64 b
**Soil fertilized with non-centrifuged waste (E) at doses of 10–40 m^3^ ha^-1^ and Apol-humus (AH; 10 L ha^-1^) and plant spraying with Stymjod (S; 5 L ha^-1^)**				
E10	0.65 bc	0.25 b	3.20 c	0.64 b
E20	0.70 de	0.28 c	3.52 d	0.69 cd
E30	0.77 g	0.34 e	3.80 ef	0.79 f
E40	0.76 g	0.33 e	3.62 de	0.74 e
E30+AH10	0.83 hi	0.38 fg	4.11 g	0.84 g
E30+AH10+S	0.90 j	0.41 h	4.43 h	0.90 h
**Soil fertilized with centrifuged waste (C) in doses of 20–60 m^3^ ha^-1^ and Apol-humus (AH; 10 L ha^-1^) and plant spraying with Stymjod (S; 5 L ha^-1^)**				
C20	0.64 ab	0.24 ab	2.99 b	0.64 b
C30	0.69 cde	0.30 cd	3.44 d	0.73 de
C40	0.75 fg	0.32 de	3.60 de	0.79 f
C50	0.71 ef	0.28 c	3.49 d	0.72 de
C60	0.66 bcd	0.25 b	3.13 bc	0.67 bc
AH10+C40	0.82 h	0.37 f	3.98 fg	0.83 fg
AH10+C40+S	0.87 ij	0.40 gh	4.38 h	0.89 h


*The data marked with the same letters within a column are not significantly different, according to Newman–Keuls multiple range test at an alpha level of 0.05.*

Enzymatic activity in plants grown in field were slightly higher in all experimental variants than in the pot experiments, however, the dependencies between doses of waste and results of enzyme activity were similar in pots and field, similarly as it was also found between waste doses and maximum PSII efficiency (Figure 1 and Tables 6, 7).

**FIGURE 3:**
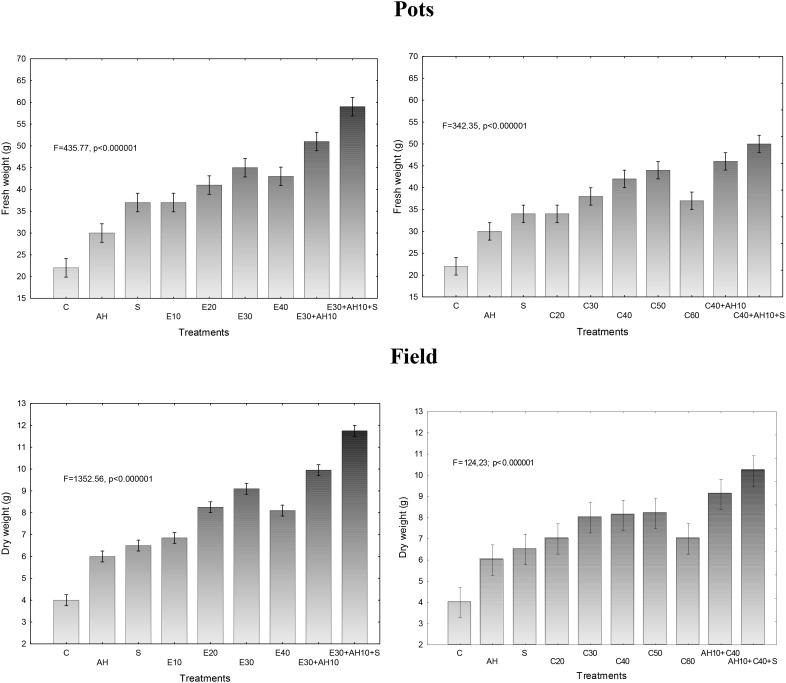
Final fresh and dry weight of one sorghum plant cultivated in pots fertilized with liquid waste from a biomass biodigestion to methane, non-centrifuged (E 10–40 m^3^ ha^-1^) and centrifuged (C 20–60 m^3^ ha^-1^), Apol-humus (AH; 10 L ha^-1^) and Stymjod (S; 5 L ha^-1^).

**FIGURE 4:**
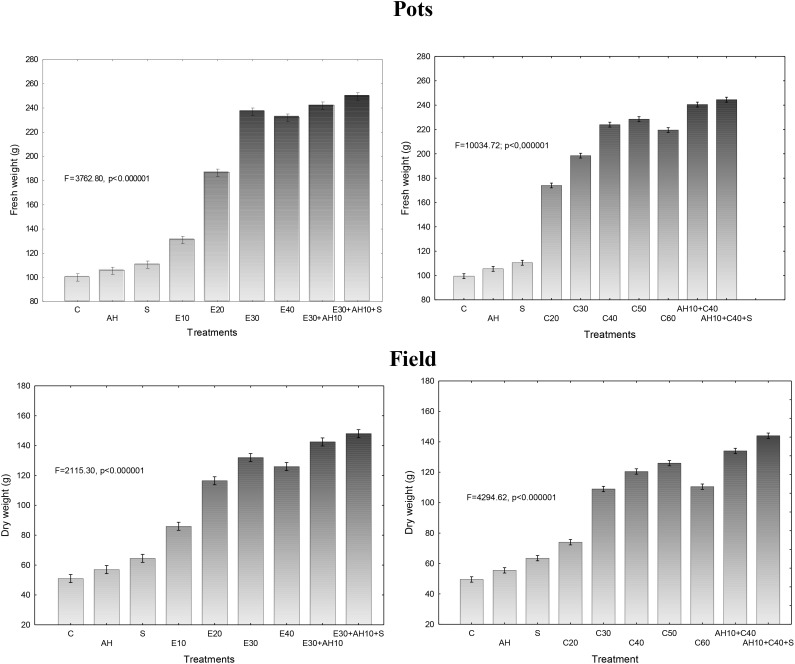
Final fresh and dry weight of one sorghum plant cultivated in field and fertilized with liquid waste from a biomass biodigestion to methane, non-centrifuged (E 10–40 m^3^ ha^-1^) and centrifuged (C 20–60 m^3^ ha^-1^), Apol-humus (AH; 10 L ha^-1^) and Stymjod (S; 5 L ha^-1^).

The applied fertilizers increased biomass yield in a degree depending on the dose of waste and the biostimulants used. Relationship between fresh or dry biomass yield and doses of waste and biostimulants were similar as shown in previous assessments. The applied non-centrifuged and centrifuged waste increased more fresh and dry biomass yield. Their doses of 30–40 m^3^ ha^-1^ and 40–50 m^3^ ha^-1^, respectively and supplemented with biostimulants were the most effective, similarly as it was shown by measurements of maximal PSII efficiency and other studies (Figures 1, 3, 4).

**FIGURE 5:**
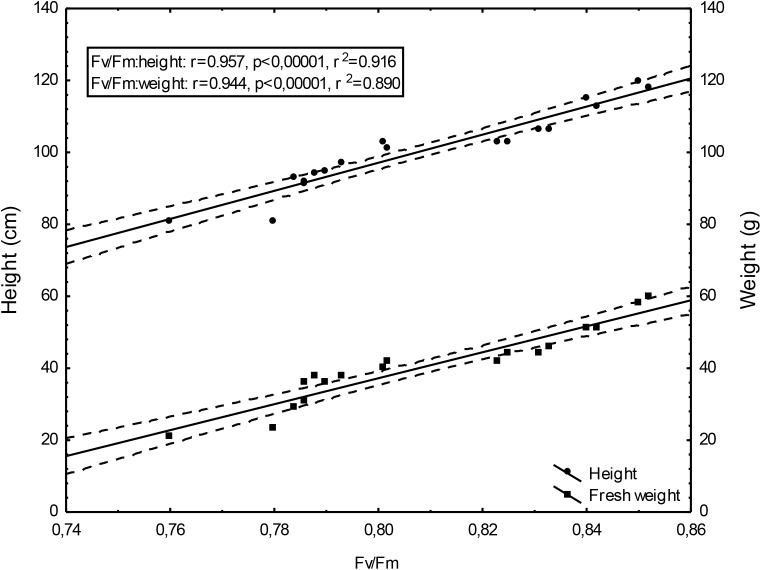
Height and fresh weight regressions against Fv/Fm: height = –215.27 + 390.48Fv/Fm and weight = –251.40 + 360.79Fv/Fm; 0,95 C.L. (Confidence limit).

**FIGURE 6:**
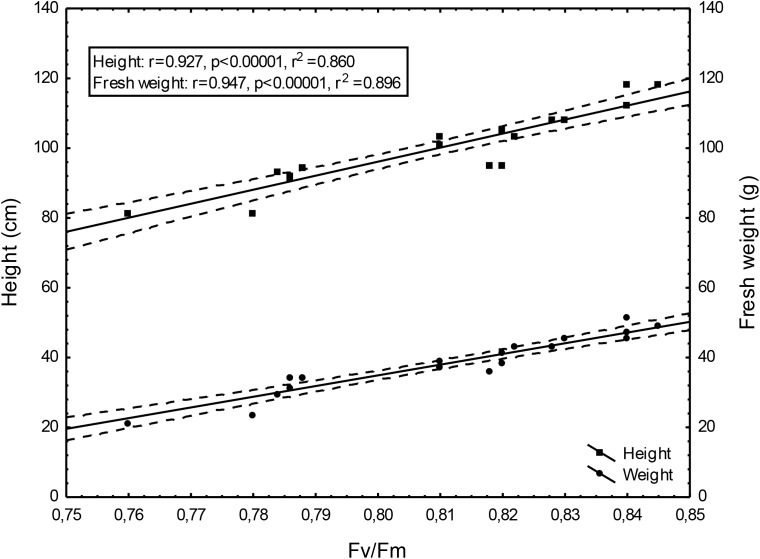
Height and fresh weight regressions against Fv/Fm: Height = –225.22 + 401.68Fv/Fm; fresh weight = –210.50 + 306.77Fv/Fm; 0,95 C.L.

**FIGURE 7:**
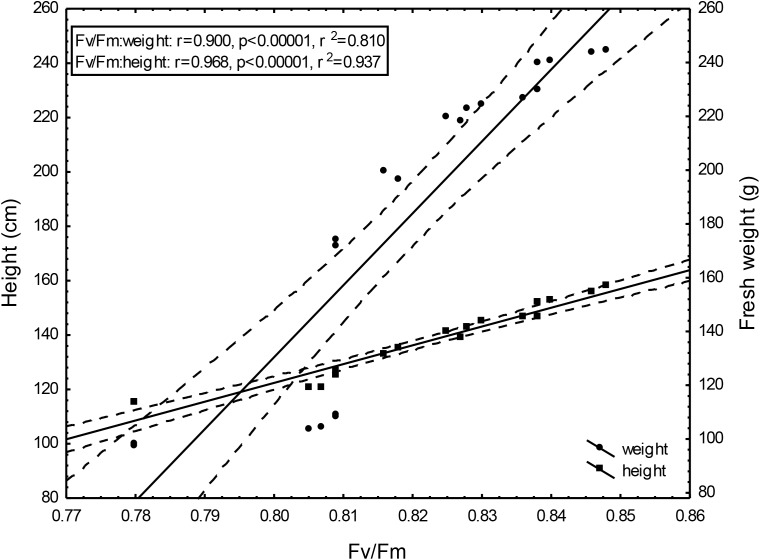
Height and fresh weight regressions against Fv/Fm: Fresh weight = –1984.95 + 2645.99Fv/Fm; 0.95 C.L. Height = –430.44 + 691,05x; 0.95 C.L.

Changing Fv/Fm values depending on the doses of un-centrifuged and centrifuged waste from a biomass biodigestion to methane used were strictly correlated with the height and mass of Sorghum plants (Figures 5–7).

Research assessing the content of macro- and micronutrients in leaves and the energy value of plants was carried out only in field conditions, due to the greater interest of energy biomass producers in them than in pot tests. The leaves of plants fertilized with the studied waste contained, in most cases, similar amounts of macro- and microelements as the non-fertilized ones, as shown in Table 8. The wastes applied to soil and supplemented with Apol-humus and Stymjod did not affected the energy value of plants and reduced only the ash content in them (Table 8).

**Table 8:** Content of macro- and microelements in leaves of sorghum plants grown in field in non-fertilized soil (Control) and that fertilized with the non-centrifuged waste from a biomass biodigestion to methane at a dose of 40 m^3^ ha^-1^ (E40) and parameters of the energy value of sorghum plants grown in field in un-fertilized soil (Control) and that fertilized with the non-centrifuged waste from a biomass biodigestion to methane at a dose of 30 m^3^ ha^-1^, Apol-humus (15 L ha^-1^) and additionally foliar spraying with Stymjod 1.5% (5 L ha^-1^).

Soil fertilization	N	P	K	Ca	Mg	Na	S.SO_4_	Fe	Mn	Cu	Zn	B
	
	[%]	[mg kg^-1^ d.w.]										
Control	2.00	4786	20490	7725	2584	126	1424	297	20,0	13.5	32.3	18.9
E 40	2.04	5146	19960	9457	3022	128	1428	311	19.9	14.4	38.2	19.6

**Evaluated properties**			**Research method**			**Unit of measure**		**Plants not fertilized**			**Plants fertilized**	

Analytical state	Analytical humidity		PN-G-04511;1980			[%]		5.21			6.40	
	Ash		PN-ISO 1171:2002			[%]		10.13			7.50	
	Heat of combustion		PN ISO 1928:2002			[kJ kg^-1^]		16631			17186	
Working state	Transient humidity		PN-G-04511;1980			[%]		50.88			49.40	
	Total humidity		PN-G-04511;1980			[%]		53.44			52.64	
	Ash		PN-ISO 1171:2002			[%]		4.98			3.80	
	Calorific value		PN ISO 1928:2002			[kJ kg^-1^]		6373			6897	


## Discussion

To the best of our knowledge, the investigation concerning the suitability of the maximum PSII efficiency, as the marker indicating vigor, growth, energetic value and physiological activity of sorghum fertilized with wastes from a biomass biodigestion to methane, in a distillery integrated with a biogas plant using corn grains as substrate, have not previously been reported. The performed studies demonstrate that the maximum PSII efficiency, as given by F_v_/F_m_ in dark-adapted leaves of sorghum plants, can be used as a non-destructive marker for defining, the degree of plant development and similarly as other studied tests (gas exchange, index of chlorophyll content and activity of selected enzymes) can indicate the plant vigor, and physiological activity. Changing Fv/Fm values depending on the doses of un-centrifuged and centrifuged waste from a biomass biodigestion to methane used were strictly correlated with the height and mass of Sorghum plants. The linear regression equation makes it possible to predict the yields of sorghum plants based on Fv/Fm. This relationship is very tight. The study exhibit the usefulness of F_v_/F_m_ in evaluation of impact of the waste from a corn grain biodigestion to methane on development and physiological activity of sorghum plants, thus showing their potential as an organic fertilizers. Obtained results indicate that F_v_/F_m_ acts as a stable metric for measuring the impact of waste fertilization on the maximal quantum yield of PSII. Additionally, this parameter responded to the waste doses positively, allowing easy interpretation for practitioners. The same was stated in Banks (2018) studies in which the F_v_/F_m_ responded to drought stress negatively. The found dependences in maximal efficiency of PSII, similarly as these in other laborious studies, closely reflected the effect of fertilization with various doses of waste, biostimulants, cultivation conditions and soil fertility on plant height, biomass yield, gas exchange and enzymatic activity and ash content in sorghum plants, which were determined in separate labor-intensive tests. These dependencies were observed independently these plants have been cultivated in peat substrate in pots or in podzolic soil in field, which showed other fertility, and under different climate in Central and North Poland. The changes in photochemical efficiency of PSII in relation to fertilization methods and sorghum growth, confirmed in other investigations, demonstrated positive impact of all studied waste doses on plant development and showed that the non-centrifuged waste is more effective in growth enhancement than the centrifuged one used in the same dose. Their application in doses of 30 m^3^ ha^-1^ and 40–50 m^3^ ha^-1^ respectively, were most efficient. These changes in F_v_/F_m_ measurements were not in line with chemical analyze results showing that plant fertilization with the waste, did not increased macro- and microelement content in leaves but it decreased only ash content in burnt sorghum biomass, which is important when these plants are produced for energetic purposes. The obtained results confirm research of Shool and Shamshiri (2014) who demonstrated the enhanced F_v_/F_m_ by improved nutritional status of the pistachio seedlings under four water regimes, especially by increasing content of the available phosphorus (P) element in soil. The close link between fluorescence emission and nitrogen fertilization was found also by Corp et al. (2003), Schächtl et al. (2005), Cendrero-Mateo et al. (2016), and Camino et al. (2018). This findings, that chlorophyll is the most widely used proxy for N content were also confirmed by research performed by Herrmann et al. (2010), Homolová et al. (2013), and Camino et al. (2018). In studies of Kalaji M.H. et al. (2014) on maize and tomato, the deficiencies of individual nutrients affected the photochemical processes of photosynthesis, as it was demonstrated by a complex of parameters derived from chlorophyll a fluorescence transient recorded *in vivo*. They stated that early detection of nutrient deficiency based on chlorophyll fluorescence data might be useful in particular species. Hamann et al. (2018) indicated that in young apple trees of two cultivars submitted to water restriction regimes fluorescence-based indices, related to chlorophyll content and nitrogen balance, promised to be a useful non-destructive tool to estimate physiological status of plants.

Studies suggest that the maximal PSII efficiency can be also used as indicator of plant health. In the conducted research, the value of this parameter grew, while when the percentage of all eight isolated pathogenic fungi in relation to the total number of isolates and the percentage of infected plants compared to controls decreased under the influence of all doses of fertilization. These found dependencies, demonstrating a lowest infection of plants showing simultaneously the highest maximal PSII efficiency, confirm usefulness of this chlorophyll fluorescence parameter in plant health status evaluation. The beneficial effect of fertilization on plant health is widely described in the literature. Possibility to use maximum PSII efficiency as indicator of plant health results from the fact that the increasing leaf colonization by pathogenic fungi damages progressively the photosynthetic system, whose activity can be assessed by F_v_/F_m_, chlorophyll content, gas exchange and other assessments. This is in line with research of Mandal et al. (2009) who demonstrated that downy mildew induced a reduction of chlorophyll content and PSII activity in *Plantago ovata*. According to Zhori et al. (2015), the non-invasive and rapid measurement of chlorophyll fluorescence induction allows characterizing the photosynthetic capacity of healthy and infected plants and of parts of them directly in the field. This test is highly sensitive not only concerning infection, but also toward other local environmental influences, as it was stated in *Euphorbia cyparissias* research. Chlorophyll a fluorescence has been satisfactorily used also for monitoring leaf health status in lamb’s lettuce (Ferrante and Maggiore, 2007).

The obtained results indicate that maximum PSII efficiency may be an rapid and not-destructive marker of sorghum plant vigor and can be profitable in fast evaluation of plant reactions to waste from a corn grain biodigestion to methane. It can be alternative or supplementary test in relation to labor-intensive and destructive studies of enzymatic activity or laboratory evaluation of chlorophyll content, which were useful in assessing the beneficial effects of various treatments on growth and physiological activity of plants. What’s more, this test is more useful in agriculture because it shows the total reaction of plants to specific treatments while others, such as chemical analysis, only results of some phenomena occurring in the tissues. In the performed research, the used maximal PSII efficiency measurements were comparable to activity of several enzymes (acid and alkaline phosphatase, RNase, dehydrogenase), index of chlorophyll content and gas exchange in leaves (net photosynthesis, transpiration, stomatal conductance and intercellular CO_2_ concentration), which were useful in assessment of physiological activity in plants and were recommended to be a markers of plant development. It was shown in research on China aster and tomato (Badek et al., 2014, 2016) and in studies showing stimulatory impact of algae on Virginia fanpetals, corn and willow growth and biostimulants on apple seed germination (Grzesik et al., 2009; Grzesik and Romanowska-Duda, 2014, 2015; Grzesik et al., 2015; Grzesik et al., 2017a,b) and sewage sludge on energy plants biomass yield (Romanowska-Duda et al., 2010). It is believed that maximum PSII efficiency measurement can be used to monitor parameters that depend on the content and activity of chlorophyll, and thus it can be an indicator of plant growth, gas exchange, activity of certain enzymes, biomass yield and the amounts of elements which depends on the functioning photosynthetic apparatus. The limiting factor of this parameter application for monitoring plant development can be a low temperature and insufficient nutrient supplementation that negatively affect the activity of the photosynthetic apparatus activity (Ostrem et al., 2018). F_v_/F_m_ can be affected by numerous environmental factors, such as high-light, extreme temperature, CO_2_, drought, and can change diurnally. Too high light and temperature are considered to be the main factors responsible for the decrease of F_v_/F_m_ at midday in terrestrial plants and in macrophytes. These changes are caused by either down-regulation or photodamage of photosystem II as a response to high irradiances (Jiang et al., 2018). In the conducted research too high temperature (well above 30°C in sun) and strong insolation and a too low air humidity (about 50%) could limit the activity of the photosynthetic system, which may cause the lower values of gas exchange parameters and photochemical efficiency of PSII (Banks, 2018). Drought is damaging when combined with other stressors, including high light intensities which cause an imbalance in the reaction centre leading to oxidative damage and photoinhibition (Goltsev et al., 2012).

Suggestions to use a maximal efficiency of PSII as a marker of sorghum growth, under influence of waste and biostimilants, are in line with research of Jalink et al. (1998) and Górnik et al. (2013) who proposed to use it in *Brassica oleracea* and coriander seeds as a non-destructive indicator for their maturity, taking into consideration that degreening process is directly related with the chlorophyll content in seed coats, which was monitored by the studied chlorophyll fluorescence parameter. Dayan et al. (2012) suggested chlorophyll fluorescence as a marker for herbicide mechanisms of action, while Silva et al. (2014) as an indicator of cellular damage by glyphosate herbicide in *Raphanus sativus* L. plants. Żebrowska and Michałek (2014) stated, that considerably differentiated phenotypic values of chlorophyll fluorescence parameters, including F_v_/F_m_, observed in the analyzed two cultivars of strawberry, were resulted from the specific response of photosynthetic apparatus, dependent on genotype to environmental conditions.

The found possibility to use maximal efficiency of PSII for rapid assessment of plant reactions to studied fertilizers become more important because amount of waste, produced by distilleries integrated with biogas plants, is rapidly growing around the world and thus its management and preferably ecological use as fertilizers for plant crops, have become one of important issues developed in the world. The tested non-centrifuged waste contained more macro- and microelements, essential for plant growth, than the centrifuged one in the unit of volume. The use of non-centrifuged waste in plant production is more justified since the costly centrifugation process is eliminated. Moreover, it contains more macro- and micronutrients thus slightly lower doses can be used to stimulate plant growth as compared to the centrifuged waste. All these measurements and findings under different climate conditions of central and northern Poland indicate, that the non-centrifuged waste from a corn grain biodigestion to methane can be used as a fertilizer in sorghum crops, solving the serious problem of its utilization and storage which is expensive and dangerous for environment. Moreover, the use of this waste in crop fertilization seems to be safer than of sewage sludge, that may contain toxic substances and thus should be refined before use in agriculture (Fang et al., 2017; Kołecka et al., 2017). The waste from corn grain biodigested to methane can be used in agriculture as fertilizers, similarly as waste from slurry digested to methane, which effectively reduce greenhouse gas emissions (Nakamura et al., 2014). However, the contents of nutrients in the studied waste was smaller (depending on the macronutrient) than in cattle manure and lower than in the waste from a maize residue biodigestion to methane in a biogas plant (Alburquerque et al., 2012; Łagocka et al., 2016), both recommended also as natural fertilizers in agriculture.

Demonstrated by maximal efficiency of PSII and other applied assessment tests, the stronger stimulating impact of the combined treatments on plant development could be caused by macro- and microelements contained in waste and by the presence of humic acids and chitosan polymers in Apol-humus, and by iodine, humic acids and nutrients in Stymjod, which additionally increased plant health, as their producers indicate. The chemical structure and stimulating impact of humic acids on plant development under normal and stress conditions was well described in literature (Calvo et al., 2014; Fahramand et al., 2014). The positive effect of chitosan on plant health, physiological activity and growth under different stress conditions was demonstrated in vines (Górnik et al., 2008) and in several other horticulture plants (Pichyangkura and Chandchawan, 2015; Bonilla and Sobral, 2016; Bistgani et al., 2017; Mukta et al., 2017; Pereira et al., 2017; Muxika et al., 2017). Kashyap et al. (2015) presented the positive impact of chitosan on production and protection of a wide number of crops and methods of its application in sustainable agriculture. The positive impact of Stymjod, applied alone and together with waste, on sorghum could be caused not only by, humic acids and elements but also by iodine. Iodine had the positive impact on the cyto-morphological changes in tomato and cabbage plants, as it was discovered by Jeznach (2011) after application of Biojodis, which was the preliminary form of Stymjod, contained similar elements, but was less absorbed by plants. Application of Biojodis to plants increased diameter of phloem and xylem tissues. Moreover, the well-formed single-layer epidermis bordered directly with one layer of collenchyma’s cells, and the parenchyma tissue greater than in control cells, was observed. Application of iodine resulted also in the predominance of open stomata which could increase gas exchange in leaves. Iodine stimulated also the physiological processes in cabbage, which were more resistant to stressed field conditions and increased the quantity of several macro- and microelements in leaves (Jeznach, 2011). In the stored carrot roots, not fertilized in field with N, treatment with iodine increased P, K, and Ca content and reduced Fe accumulation (Smoleń et al., 2011). Application of Biojodis also alleviated the negative influence of unfavorable temperature and drought on growth and physiological activity of corn plants (Piotrowski et al., 2016) and Virginia fanpetals (Grzesik et al., 2009). In lettuce, the winter and summer application of iodate (IO_3_^-^) or iodide (I^-^) increased quantity of these elements in leaves (Voogt et al., 2010). The mentioned processes found in vegetable crops, could stimulate also the growth of sorghum by increasing transportation of nutrients from reached by waste soil through the wider xylem cells, increased the plant growth and enhanced photosynthetic system activity, assessed by maximal efficiency of PSII, however, research in this area was not carried out.

## Conclusion

The performed studies demonstrate that the maximum PSII efficiency, as given by F_v_/F_m_ in dark-adapted leaves of sorghum plants, can be used as a non-destructive marker for defining, the degree of sorghum plant development. Similarly as other studied tests (gas exchange, index of chlorophyll content, activity of selected enzymes, mycological and chemical analyzes) it can be used to monitor the plant growth, physiological activity, infestation by pathogenic fungi, and ash content in burnt sorghum biomass. The presented maximum PSII efficiency assessments, confirming other physiological test measurements, shows prospects of ecological use of the waste from a corn grain biodigestion to methane in sorghum crops as fertilizers, together with the new generation soil improver Apol-humus and the nano-organic-mineral fertilizer, Stymjod, as the alternative to artificial fertilizers which pollute the environment. Thus, the maximum PSII efficiency can be seen as useful marker helping management of the studied waste as economically and environmentally friendly natural stimulants, if they are used in defined doses and in agreement with national, legal regulations on the safe application of fertilizers. Confirmed that the linear regression equation makes it possible to predict the yields of sorghum plants based on Fv/Fm. This relationship is very tight.

## Author Contributions

ZR-D carried out the experiments, processed the experimental data and wrote part of the research dealing with maximal efficiency of PSII, and enzyme activity. MG conducted the research, processed the experimental data, and wrote the part of article on plant development and the gas exchange. RJ conducted experiments on the health of plants, their infestation by pathogenic mycoflora and the quality and quantity of pathogenic microflora infesting plants. All authors provided critical feedback, made contributions to analysis and interpretation of data, discussed the results, contributed to the writing of the manuscript, and gave final approval of the version to be published.

## Conflict of Interest Statement

The authors declare that the research was conducted in the absence of any commercial or financial relationships that could be construed as a potential conflict of interest.
